# Tolerance Inversion for Lens Support Loads Based on Feature-Enhanced Active-Learning Gaussian Process Regression

**DOI:** 10.3390/mi17080960

**Published:** 2026-08-14

**Authors:** Jingteng Liu, Shiyu Li, Xia Kang, Songmao Xian, Ji Zhou, Junbo Liu

**Affiliations:** 1 State Key Laboratory of Optical Field Manipulation Science and Technology, Institute of Optics and Electronics, Chinese Academy of Sciences, Chengdu 610209, China; liujingteng24@mails.ucas.ac.cn (J.L.);; 2 University of Chinese Academy of Sciences, Beijing 100049, China

**Keywords:** lithographic objective, lens support load, tolerance inversion, regional peak-valley fluctuation feature, active learning, Gaussian process regression

## Abstract

Support load fluctuations in lithographic objectives can induce additional surface figure errors in lenses. Conventional Monte Carlo-based tolerance analysis is computationally expensive in high-dimensional load spaces, and surrogate models based only on raw load inputs often fail to accurately capture local-extremum responses such as peak-to-valley (PV). To address the load tolerance inversion problem under prescribed PV and root mean square (RMS) constraints, a tolerance inversion framework integrating Regional Peak-to-Valley Fluctuation Features, Active-Learning Gaussian Process Regression, and dual-metric tolerance boundary search (RPVF-ALGPR) is proposed. The framework transforms the local fluctuation information in low-order Zernike-reconstructed wavefronts into regional peak-to-valley fluctuation features and combines them with the original support loads as inputs to the GPR surrogate models. It further combines posterior-uncertainty-driven active learning to construct surrogate models for both metrics, thereby enabling the inverse determination of the critical load fluctuation boundary. One set of training results from a biconvex lens case study shows that the proposed method effectively improves PV and RMS prediction accuracy and reduces the number of samples required to reach the prescribed accuracy threshold by 35.7% compared with random sampling. The results provide a reference for support-load tolerance allocation and optomechanical stability evaluation of high-precision lenses.

## 1. Introduction

Semiconductor lithography equipment is one of the core enabling systems for microscale and nanomanufacturing. The wavefront quality, imaging resolution, and long-term stability of its projection objective directly affect the accuracy of microscale and nanoscale pattern transfer. For lithographic objectives and other high-end precision optical systems, the surface figure stability of high-precision lenses after assembly depends not only on the manufacturing accuracy of the optical elements but also on the precision positioning support structure, preload state, and assembly tolerance control [1,2,3,4]. Therefore, the lens support structure is not merely a mechanical load-bearing component, but an important optomechanical coupling element for maintaining optical performance and suppressing assembly-induced errors in micro- and nanomanufacturing equipment. To reduce self-weight sag, mitigate clamping stress, and accommodate thermal mismatch, multipoint flexible support has become a common support configuration for high-precision optical elements, especially in lithographic projection objectives where accuracy requirements are extremely stringent [5,6,7,8]. However, during actual assembly, preload errors, differences in contact stiffness, manufacturing and alignment deviations, bonding constraints, and environmental disturbances can still cause the support loads to deviate from the ideal uniform-load state, thereby inducing additional surface figure distortion and degradation of imaging quality [9,10]. It is therefore necessary to systematically investigate the quantitative relationship between support load fluctuations and surface figure errors.

To address this problem, most existing research follows the forward analysis route of applying given loads and computing surface shape responses. In early research, Shen et al. regarded the support loads as random variables, established the statistical relationship between load fluctuations and surface deformation through large-scale finite element simulations and Monte Carlo sampling, and determined the critical range of load variation using the 3σ criterion [9]. Genberg et al. integrated Monte Carlo techniques into the opto-mechanical tolerance analysis process, achieving engineering statistical tolerance evaluation based on indicators such as RMS [10]. In recent years, performance domain tolerance analysis, local tolerance analysis, and flexible design research for complex support structures have improved the tolerance design capabilities of complex optical systems from different perspectives [11,12,13]. However, the above work conceptually still has not departed from the framework of forward statistical evaluation, namely computing surface shape responses through a large number of finite element simulations combined with Monte Carlo analysis. This approach has high computational cost under conditions of high-dimensional support load space and large fluctuation ranges, making it difficult to directly meet the practical engineering need to quickly determine the allowable load fluctuation range based on optical indicator constraints.

To reduce the cost of repeated simulation, recent work introduces surrogate models and active learning into high-cost engineering analysis. Gaussian process regression and related surrogate methods perform well in small-sample, high-cost, and nonlinear problems. They can approximate complex finite element models with limited samples while naturally providing predictive uncertainty [13,14,15,16,17]. Active learning further improves sample efficiency by preferentially adding samples with high posterior variance or strong boundary sensitivity, which improves model accuracy in critical regions [18,19,20,21]. However, conventional surrogate models remain inadequate for response modeling between support loads and surface figure error in large-aperture lenses. First, support load and surface error are strongly nonlinear and coupled. In particular, peak-to-valley (PV) is highly sensitive to local surface variations [4,22]. If the model uses only the original load data as input, it is difficult to capture this extreme-response behavior in a stable way. Second, most existing studies focus on forward prediction of surface error from load input. Even with a highly accurate forward surrogate model, the allowable fluctuation range of support loads under PV and root-mean-square (RMS) constraints cannot be obtained directly. Additional boundary search or constraint evaluation is still required to inversely determine the tolerance boundary [23,24]. These issues indicate that input feature construction, surrogate modeling, and boundary inversion need to be improved together.

Based on the above background, this paper investigates a multi-point compliant support system for high-precision lenses in micro- and nano-manufacturing equipment such as lithographic objectives. To address the inverse tolerance problem of support load fluctuations under specified PV and RMS constraints, a Regional Peak-to-Valley Fluctuation Feature-enhanced Active-Learning Gaussian Process Regression framework for tolerance inversion (RPVF-ALGPR) is constructed. The framework first establishes surrogate mappings from the support loads to the low-order Zernike coefficients and extracts regional peak-to-valley fluctuation features from the low-order reconstructed wavefront, so that local surface figure fluctuation information can be incorporated into PV/RMS surrogate modeling in the form of low-dimensional features. Subsequently, posterior-uncertainty-driven active learning is used to supplement highly informative samples, and a dual-metric constrained boundary search is performed to inversely determine the allowable range of support load fluctuations. In this way, regional peak-to-valley fluctuation features, active-learning-based surrogate modeling, and tolerance boundary inversion are integrated into a unified procedure to improve the efficiency of support load tolerance inversion under limited simulation samples. This research can provide a quantitative reference for support load tolerance allocation, assembly control, and optomechanical stability evaluation of precision optical systems such as lithographic objectives.

## 2. Tolerance Inversion Method Based on Feature Enhancement and Active Learning

### 2.1. Definition of Support Load Fluctuation and Tolerance Inversion Problem

**Figure 1:**
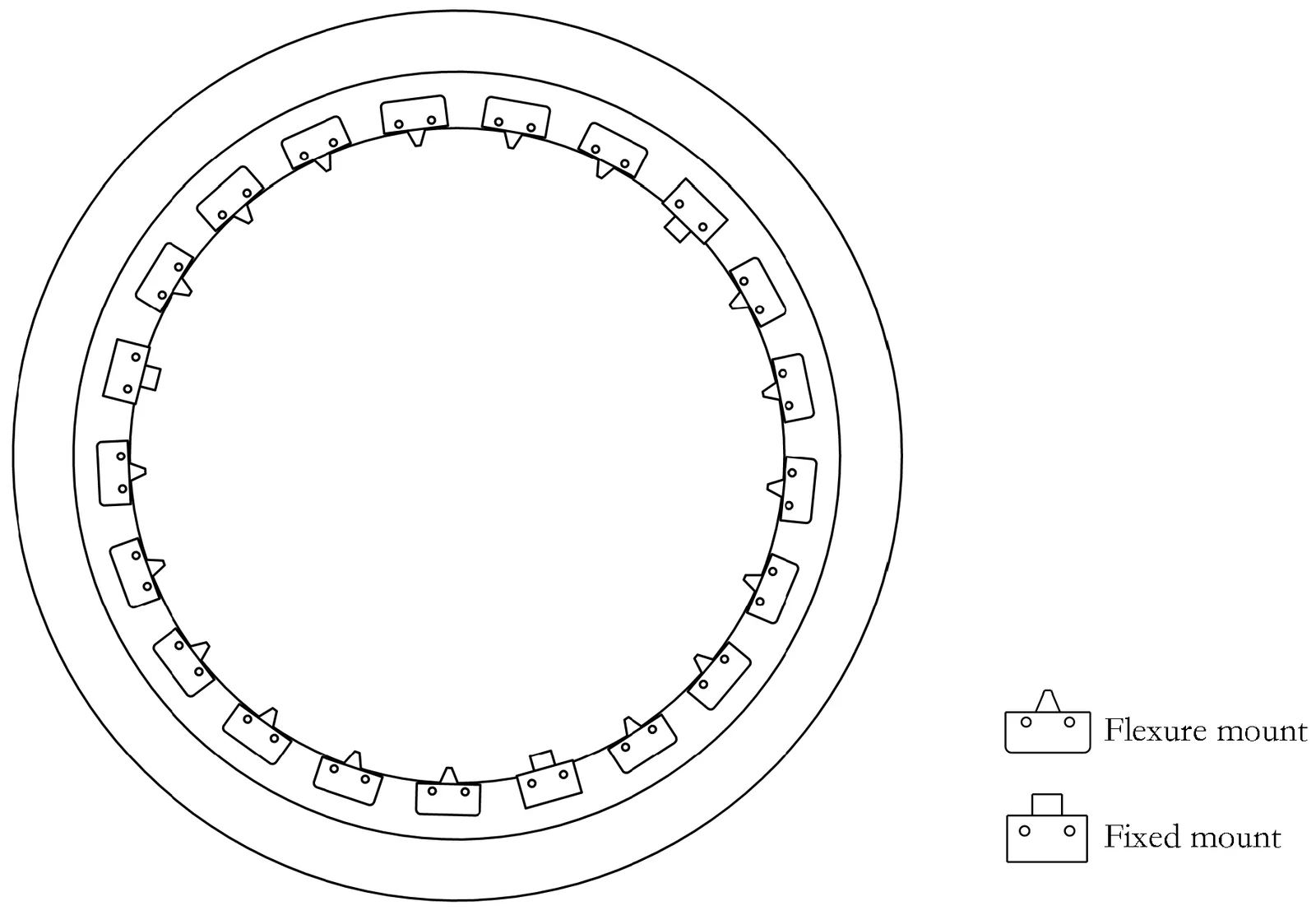
Schematic of the lens support system structure.

Lenses in high-precision optical systems, such as lithographic projection objectives, commonly adopt a support configuration that combines three fixed supports with multiple flexible supports, as shown in Figure 1. The three fixed supports are mainly used to define the spatial position of the lens, while the flexible supports share the lens weight and reduce local additional stresses caused by assembly and alignment errors. Under ideal conditions, the lens weight is carried jointly by all support points; that is, the fixed and flexible supports share the self-weight of the lens according to the same theoretical load.

Let the lens mass be *M*, the gravitational acceleration be *g*, and the number of flexible support points be *n*. Then, the lens gravity is Mg, and the total number of support points is n+3. Under the ideal uniform stress state, the theoretical support load of a single fixed support point or flexible support point can be expressed as

(1) $$F_{0}=\frac{Mg}{n+3}$$

During actual assembly process, the load deviations at different flexible support points are generally nonuniform. Some supports may carry loads higher than their theoretical values, whereas others may carry lower loads. Let the theoretical load of the *i*-th flexible support point be $F_{i}^{0}$ and the actual load be $F_{i}$, and use $x=(F_{1},\ldots ,F_{n})$ to denote the load vector composed of the actual loads of all flexible support points. To uniformly describe the overall fluctuation range of a set of flexible support actual loads relative to the theoretical loads, a normalized fluctuation amplitude Δ is introduced, which represents the maximum allowable relative deviation ratio of each flexible support point’s actual load relative to the theoretical load.

Under given Δ conditions, the feasible domain of flexible support loads can be expressed as

(2) $$\Omega \left(\Delta \right)=\left\{x=\left(F_{1},\ldots ,F_{n}\right)\;|\;\left|\frac{F_{i}-F_{i}^{0}}{F_{i}^{0}}\right|\leq \Delta ,\;F_{i}\geq 0,\;i=1,\ldots ,n\right\}$$

In Equation (2), Ω(Δ) is the feasible domain of flexible support loads under a given fluctuation amplitude. As Δ increases, the allowable deviation of the actual flexible support loads from their theoretical values becomes larger, indicating a potentially more pronounced load imbalance in the support system. This imbalance changes the boundary loading conditions of the lens and further alters the surface-normal displacement field. To evaluate the resulting surface figure response, let $w_{j}$ be the normal displacement at the *j*-th sampling node within the effective aperture, *N* be the total number of sampling nodes, and $\bar{w}$ be the mean normal displacement. The surface figure metrics RMS and PV are then defined as

(3) $$\left\{\begin{array}{c} \mathrm{RMS}=\sqrt{\frac{1}{N}\sum_{j=1}^{N}{\left(w_{j}-\bar{w}\right)}^{2}}\\ \mathrm{PV}=w_{\max}-w_{\min} \end{array}\right.$$

where RMS characterizes the overall dispersion degree of the full-aperture displacement distribution, and PV characterizes the maximum peak-valley difference of the mirror surface normal displacement field. Therefore, the forward modeling problem under support load fluctuation conditions can be expressed as the mapping relationship from the flexible support actual load vector to the dual surface shape indicators:

(4) $$x=\left(F_{1},F_{2},\ldots ,F_{n}\right)\mapsto y=\left(\mathrm{PV},\mathrm{RMS}\right)$$

In practical tolerance design, the allowable upper limits of the surface figure error, ${\mathrm{PV}}_{\mathrm{tol}}$ and ${\mathrm{RMS}}_{\mathrm{tol}}$ are usually prescribed in advance. The objective is not merely to predict the surface figure error under a given load combination but to inversely determine the maximum allowable fluctuation range of the actual flexible-support loads relative to their theoretical values while satisfying both metric constraints. Accordingly, the tolerance inversion objective can be formulated as

(5) $$\Delta^{*}=\sup\left\{\Delta |\mathrm{PV}\left(x\right)\leq {\mathrm{PV}}_{\mathrm{tol}},\;\mathrm{RMS}\left(x\right)\leq {\mathrm{RMS}}_{\mathrm{tol}},\;\forall x\in \Omega \left(\Delta \right)\right\}$$

where $\Delta^{*}$ denotes the critical fluctuation boundary allowed for the actual flexible-support loads relative to their theoretical values under the PV and RMS constraints. The problem can therefore be summarized as follows: under a limited number of simulation samples, a forward surrogate model is established to relate support load fluctuations to surface figure errors, and this model is then used to inversely determine the maximum allowable load fluctuation range satisfying the prescribed surface figure constraints.

### 2.2. RPVF Construction

Support load fluctuations alter the spatial distribution of the surface-normal displacement field of the lens. Among the surface figure metrics, RMS mainly reflects the overall dispersion of the full-aperture response, whereas PV is more sensitive to the locations and magnitudes of local peaks and valleys. Therefore, if only the support load vector x is used as the surrogate model input, the model needs to indirectly infer the spatial evolution pattern of local extrema from load perturbations, which makes accurate PV prediction difficult under limited training samples. To address this issue, a Regional Peak-to-Valley Fluctuation Feature (RPVF) is introduced in addition to the original load inputs. The RPVF is defined as the peak-to-valley variation in the low-order Zernike-reconstructed wavefront within each local region. It characterizes regional surface fluctuations along different azimuthal directions and thereby improves the model’s ability to predict local peak-to-valley responses. In the proposed framework, the RPVF and the original support loads jointly constitute the enhanced input used for subsequent PV/RMS surrogate model training and tolerance boundary search. A Gaussian Process Regression (GPR) surrogate mapping is first constructed from the support load vector x to the low-order Zernike coefficients. For the *j*-th Zernike coefficient, the mapping is expressed as

(6) $${\hat{z}}_{j}\left(x\right)=G_{j}\left(x\right)\;j=1,2,\ldots ,J$$

where $G_{j}$ is the GPR submodel corresponding to the *j*-th Zernike coefficient, ${\hat{z}}_{j}\left(x\right)$ is the predicted coefficient, and *J* is the number of Zernike truncation terms. Based on the predicted coefficients, the low-order approximate wavefront can be reconstructed as

(7) $${\hat{w}}_{J}\left(\rho ,\theta ;x\right)=\sum_{j=1}^{J}{\hat{z}}_{j}\left(x\right)Z_{j}\left(\rho ,\theta \right)$$

where (ρ,θ) are the normalized aperture coordinates, $Z_{j}$ is the *j*-th Zernike basis function, and ${\hat{w}}_{J}$ is the approximate wavefront reconstructed from the predicted coefficients. Low-order Zernike reconstruction captures the main spatial variation of the lens surface response with relatively few parameters, providing a basis for subsequent local feature extraction.

**Figure 2:**
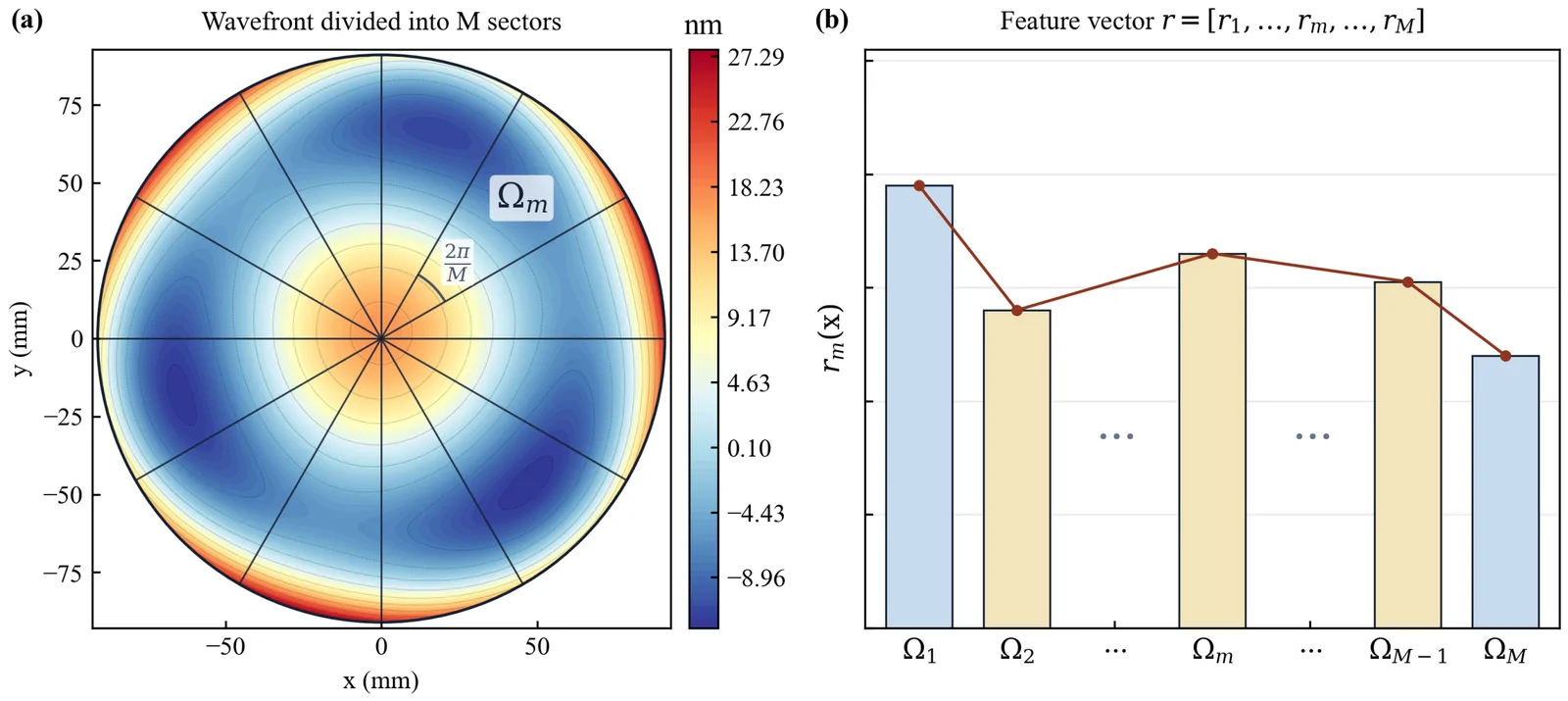
Schematic of regional peak-to-valley fluctuation feature construction: (**a**) The low-order wavefront reconstructed based on predicted Zernike coefficients, with the effective aperture divided into *M* equal sector regions $\Omega_{m}$; (**b**) The RPVF vector $r=[r_{1},\ldots ,r_{m},\ldots ,r_{M}]$ composed of the fluctuation amplitudes of each sector region; the bar colors are used only to distinguish adjacent feature components, and the connected markers indicate the ordered RPVF vector.

As shown in Figure 2a, after reconstructing the wavefront, the effective circular aperture is divided into *M* equal sector regions $\Omega_{m}$, each subtending a central angle of 2π/M. The peak-to-valley variation in the reconstructed wavefront within each sector is then computed and defined as the *m*-th component $r_{m}$ of the RPVF:

(8) $$r_{m}\left(x\right)=\max_{p\in \Omega_{m}}{\hat{w}}_{J}\left(p;x\right)-\min_{p\in \Omega_{m}}{\hat{w}}_{J}\left(p;x\right)\;m=1,2,\ldots ,M$$

where *p* is a discrete sampling node within region $\Omega_{m}$, and $r_{m}\left(x\right)$ is the reconstructed wavefront fluctuation amplitude within the *m*-th sector region. The RPVF compresses local peak-to-valley variations along different azimuthal directions into a set of low-dimensional scalar features, thereby preserving spatial information while avoiding the direct use of high-dimensional displacement-field data. Concatenating the RPVF vector $r\left(x\right)=[r_{1}\left(x\right),\ldots ,r_{M}\left(x\right)]$ with the raw support load vector constructs the enhanced input vector:

(9) $$\tilde{x}=\left[x;r_{1}\left(x\right),r_{2}\left(x\right),\ldots ,r_{M}\left(x\right)\right]$$

where $\tilde{x}$ contains both the support load information and the local surface fluctuation information. Compared with directly using full-field displacement data, this enhanced input has lower dimensionality. Compared with using only raw load vector, it provides the surrogate model with spatial response features that are more directly related to local peak-to-valley variations in PV.

In practical implementation, the low-order Zernike coefficients of each training sample are obtained by fitting the finite element displacement field, and the corresponding Zernike-coefficient GPR submodels are then trained. For the validation set, test set, and candidate points used in boundary inversion, the RPVF features are generated by the trained GPR submodels rather than from the true displacement fields of the samples to be predicted. This avoids information leakage during model evaluation and boundary search.

### 2.3. GPR Model and Active Learning

The construction of RPVF and the prediction of surface figure metrics can both be formulated as nonlinear regression problems under limited training samples. To maintain a consistent modeling framework, GPR is used to construct two types of surrogate mappings. The first maps the actual support load vector x to the low-order Zernike coefficients ${\hat{z}}_{j}\left(x\right)$, which are used to generate the regional fluctuation features. The second maps the enhanced input $\tilde{x}$ to the PV and RMS surface figure metrics. After input enhancement, the prediction of surface figure metrics is therefore treated as a regression problem from $\tilde{x}$ to the PV/RMS responses. For the standardized response function $f(\tilde{x})$, GPR assumes the following Gaussian process prior:

(10) $$f\left(\tilde{x}\right)∼\mathrm{GP}\left(0,k_{\theta }\left(\tilde{x},{\tilde{x}}^{\prime }\right)\right)$$

where $k_{\theta }\left(\cdot ,\cdot \right)$ is the covariance kernel function, $\tilde{x}$ and ${\tilde{x}}^{\prime }$ represent any two enhanced input samples, and θ are the kernel function hyperparameters. Considering that different feature dimensions in the enhanced input may have different impact scales on the surface shape response, an anisotropic radial basis function kernel is adopted:

(11) $$k_{\theta }\left(\tilde{x},{\tilde{x}}^{\prime }\right)=\sigma_{f}^{2}\exp\left[-\frac{1}{2}\sum_{d=1}^{D}\frac{{\left({\tilde{x}}_{d}-{\tilde{x}}_{d}^{\prime }\right)}^{2}}{\ell_{d}^{2}}\right]$$

where $\sigma_{f}^{2}$ is the signal variance, $\ell_{d}$ is the length scale corresponding to the *d*-th dimension input feature, and *D* is the dimension of the enhanced input $\tilde{x}$. Before model training, both the input features and output responses are standardized, and the kernel hyperparameters are optimized by maximizing the log marginal likelihood. PV and RMS are modeled as two independent responses to reduce the influence of their differences in numerical scale and response behavior on model training, and to allow their posterior uncertainties to be evaluated separately during active learning.

When finite element simulations are computationally expensive and only a limited number of samples are available, the prediction accuracy of the surrogate model is strongly affected by the coverage of the training samples. Active learning can improve the model in poorly represented regions by adding a small number of highly informative samples. Compared with direct modeling based on a large number of samples, this strategy improves modeling efficiency under limited-sample conditions. Since the posterior variance of GPR reflects the prediction uncertainty at candidate load points, it is used as the sampling criterion, and candidates with higher uncertainty are prioritized for enrichment.

First, uniform random sampling is used within the preset load fluctuation range to generate the candidate load set $C_{x}$, where all candidates satisfy the feasible-domain constraint defined in Equation (2). For each candidate load $x\in C_{x}$, the Zernike-coefficient GPR models are used to generate the RPVF features and construct the enhanced input $\tilde{x}$. The posterior variances are then calculated using the PV and RMS surrogate models. Since both PV and RMS are involved in the subsequent tolerance boundary determination, the combined posterior uncertainty is defined as

(12) $$U\left(x\right)=\sigma_{\mathrm{PV}}^{2}\left(\tilde{x}\right)+\sigma_{\mathrm{RMS}}^{2}\left(\tilde{x}\right)\;x\in C_{x}$$

where $\sigma_{\mathrm{PV}}^{2}\left(\tilde{x}\right)$ and $\sigma_{\mathrm{RMS}}^{2}\left(\tilde{x}\right)$ are the posterior variances of the PV and RMS surrogate models at the candidate enhanced input, respectively. In each active learning iteration, the candidate sample with the largest combined uncertainty is selected for finite element simulation:

(13) $$x^{*}=\arg\max_{x\in C_{x}}U\left(x\right)$$

After the new sample is simulated, it is added to the training set, and its corresponding low-order Zernike coefficients, PV and RMS are extracted. The Zernike-coefficient GPR models and the GPR models for the PV and RMS responses are then updated simultaneously. In the next iteration, a new candidate load set is regenerated, and the enhanced inputs and posterior uncertainties of the candidate samples are recalculated before continuing sample selection. One new sample is added in each iteration until the preset sample budget is reached.

### 2.4. Tolerance Boundary Inversion Under Dual-Indicator Constraints

For practical tolerance design, the primary concern is the maximum allowable deviation of the actual flexible-support loads from their theoretical values under prescribed PV and RMS tolerance limits. To estimate this critical boundary, a bisection search strategy is adopted to locate the maximum allowable fluctuation $\Delta^{*}$ within the preset fluctuation range. First, a fixed set of test samples is generated in the normalized load fluctuation space using Latin hypercube sampling. Then, for each candidate fluctuation amplitude Δ, this fixed sample set is scaled by Δ to obtain the corresponding test load set $C_{x}\left(\Delta \right)$. For any test load $x\in C_{x}\left(\Delta \right)$, the enhanced input $\tilde{x}$ is first constructed, then the corresponding PV and RMS are predicted by the enhanced GPR surrogate model, and whether this candidate fluctuation amplitude satisfies the performance constraints is determined based on the prediction results.

To account for both PV and RMS surface figure constraints, a constraint function is defined for each test load:

(14) $$r\left(x\right)=\max\left\{\frac{\hat{\mathrm{PV}}\left(\tilde{x}\right)}{{\mathrm{PV}}_{\mathrm{tol}}},\frac{\hat{\mathrm{RMS}}\left(\tilde{x}\right)}{{\mathrm{RMS}}_{\mathrm{tol}}}\right\}-1$$

When r(x)≤0, this test load satisfies the dual-indicator constraints; when r(x)>0, at least one surface shape indicator exceeds the corresponding tolerance upper limit. This function can also be used to rank the risk levels of different loads. The larger r(x) is, the closer or more likely this load is to exceed the PV or RMS tolerance constraints.

For a certain candidate fluctuation amplitude Δ, the feasibility of this fluctuation level is evaluated using the maximum value of the constraint function over the test set:

(15) $$g\left(\Delta \right)=\max_{x\in C_{x}\left(\Delta \right)}r\left(x\right)$$

where g(Δ) denotes the maximum value of the constraint function over the test set. When g(Δ)≤0, all loads in the test set are predicted by the surrogate model to satisfy the constraints. When g(Δ)>0, at least one sample in the test set is predicted to violate the tolerance limits.

Based on this criterion, a bisection algorithm is used to search for the critical fluctuation boundary within the prescribed fluctuation range. At each iteration, $g(\Delta_{\mathrm{mid}})$ is evaluated at the midpoint $\Delta_{\mathrm{mid}}$ of the current interval, and the search interval is updated according to its sign. If $g(\Delta_{\mathrm{mid}})\leq 0$, the lower bound is updated to $\Delta_{\mathrm{mid}}$; otherwise, the upper bound is updated to $\Delta_{\mathrm{mid}}$.This procedure is repeated until the interval width converges to the prescribed tolerance, and the resulting $\Delta^{*}$ is taken as an estimate of the critical fluctuation boundary under the surrogate-model prediction and the finite candidate sample pool.

## 3. Experimental Design and Results Analysis

### 3.1. Experimental Setup

**Figure 3:**
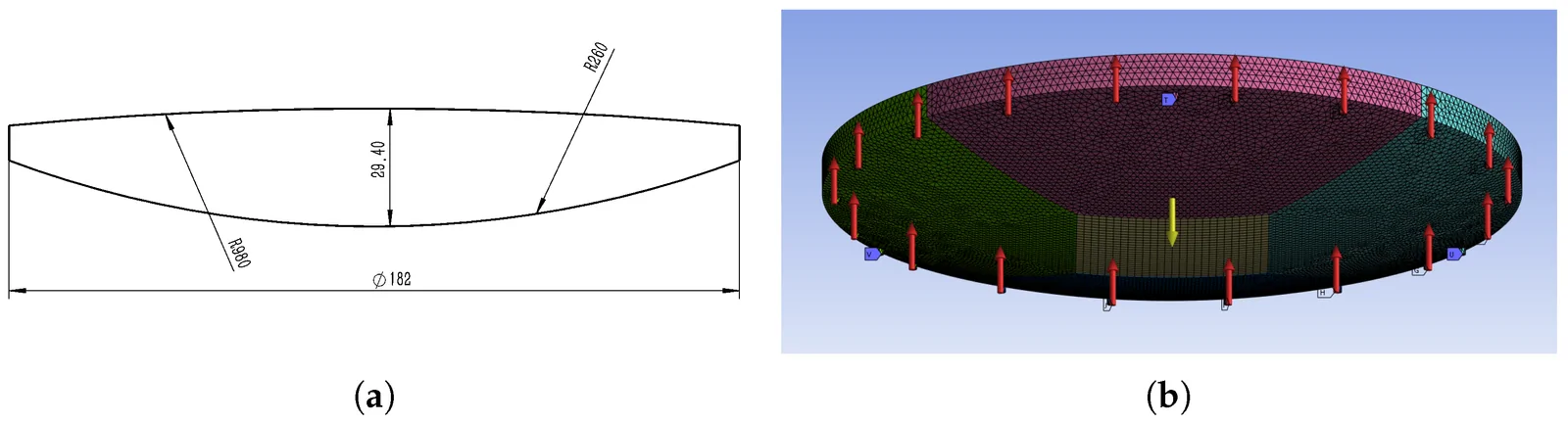
Schematic of lens geometric parameters and finite element model: (**a**) Lens geometric cross-section; (**b**) Finite element mesh and support boundary conditions, where red arrows indicate the direction of flexible support loads, blue regions indicate fixed constraints, and yellow arrows indicate the direction of gravity.

A biconvex circular glass lens is used as the research object, with lens geometric parameters and material parameters shown in Figure 3a and Table 1, respectively. The lens is supported by a combined configuration consisting of three fixed supports and 18 flexible supports, giving a total of 21 support points. The three-dimensional solid finite element model of the lens support system is shown in Figure 3b, and the mesh contains 472,334 elements. To simplify the simulation, the flexible support mechanisms are not explicitly modeled; instead, the support loads are applied as vertical concentrated forces at the 18 flexible support points. The boundary conditions of the fixed supports are imposed through the lens frame. Specifically, the outer lens frame is fixed, and the contacts between the frame and the three fixed support points are defined as bonded constraints, thereby applying full degrees-of-freedom constraints to the fixed support points. After each support-load simulation is completed, the surface-normal displacement field on the top surface of the lens is extracted and imported into Sigfit for Zernike polynomial fitting, from which the surface figure metrics PV and RMS are calculated.

According to the material parameters in Table 1 and the geometric dimensions in Figure 3a, the lens weight is calculated as Mg=12.367N. From Equation (1), the theoretical load at each support point is $F_{0}=0.5889\;N$. The target fluctuation amplitude is set to $\Delta_{t}=0.30$, corresponding to an actual load constraint of $F_{i}\in \left[0.4122,0.7655\right]\;N$ for each flexible support point.

The experiments are conducted in three stages. First, static ablation studies are performed to evaluate the effect of different input configurations on prediction performance. In this stage, 20 finite element samples are used to construct the initial surrogate model, and 40 independent finite element samples are used for performance evaluation. Second, active learning experiments are carried out within the feasible domain of the target condition $\Omega (\Delta_{t})$. The initial training set contains 20 samples, and the maximum sample budget is set to 80. In each active learning iteration, 1000 candidate load samples are generated by uniform random sampling within the preset load fluctuation range, and the sample with the largest combined posterior uncertainty is selected for finite element simulation. The random seed in this stage is set to 42. Finally, the model satisfying the prescribed PV accuracy threshold after active learning is selected. Combined with the boundary search strategy described in Section 2.4, it is used to inversely determine the critical fluctuation boundary $\Delta^{*}$ under the given tolerance constraints ${\mathrm{PV}}_{\mathrm{tol}}$ and ${\mathrm{RMS}}_{\mathrm{tol}}$. In the tolerance boundary inversion stage, 3000 fixed test samples are generated in the normalized load fluctuation space using Latin hypercube sampling, with the random seed set to 20260512. This fixed test sample set is reused for different candidate fluctuation amplitudes Δ to reduce the influence of repeated random sampling on the stability of boundary determination.

### 3.2. Feature Enhancement Effect Analysis

The predictive performance of the surrogate model depends not only on the regression model itself, but also on the representational capability of the input features and the parameters used for feature construction. To evaluate the effectiveness of the RPVF features, a sensitivity analysis is first conducted on the Zernike truncation term number *J* and the number of sector regions *M*, so as to determine the default feature construction parameters used in the subsequent experiments. Then, under the same training samples, test samples, and model settings, static ablation comparisons are performed for different input construction schemes to verify the improvement brought by the regional peak-to-valley fluctuation features in predicting PV and RMS.

The model prediction performance is evaluated using the coefficient of determination ($R^{2}$) and the root mean square error (RMSE), which are defined as

(16) $$\left\{\begin{array}{c} R^{2}=1-\frac{\sum_{i=1}^{N_{t}}{\left(y_{i}-{\hat{y}}_{i}\right)}^{2}}{\sum_{i=1}^{N_{t}}{\left(y_{i}-\bar{y}\right)}^{2}}\\ \mathrm{RMSE}=\sqrt{\frac{1}{N_{t}}\sum_{i=1}^{N_{t}}{\left(y_{i}-{\hat{y}}_{i}\right)}^{2}} \end{array}\right.$$

where $y_{i}$ and ${\hat{y}}_{i}$ represent the true value and model prediction value of test samples, respectively, $\bar{y}$ represents the mean of true test set responses, and $N_{t}$ is the number of test samples. The coefficient of determination $R^{2}$ is used to evaluate the model’s fitting ability to the variation pattern of test sample responses. The closer $R^{2}$ is to 1, the stronger the model’s capability to restore the true PV or RMS variation trend. RMSE is used to quantify the average deviation between predicted values and true values. The smaller its value, the lower the absolute prediction error of the model and the more reliable the prediction results.

#### 3.2.1. Sensitivity Analysis of RPVF Parameters

**Figure 4:**
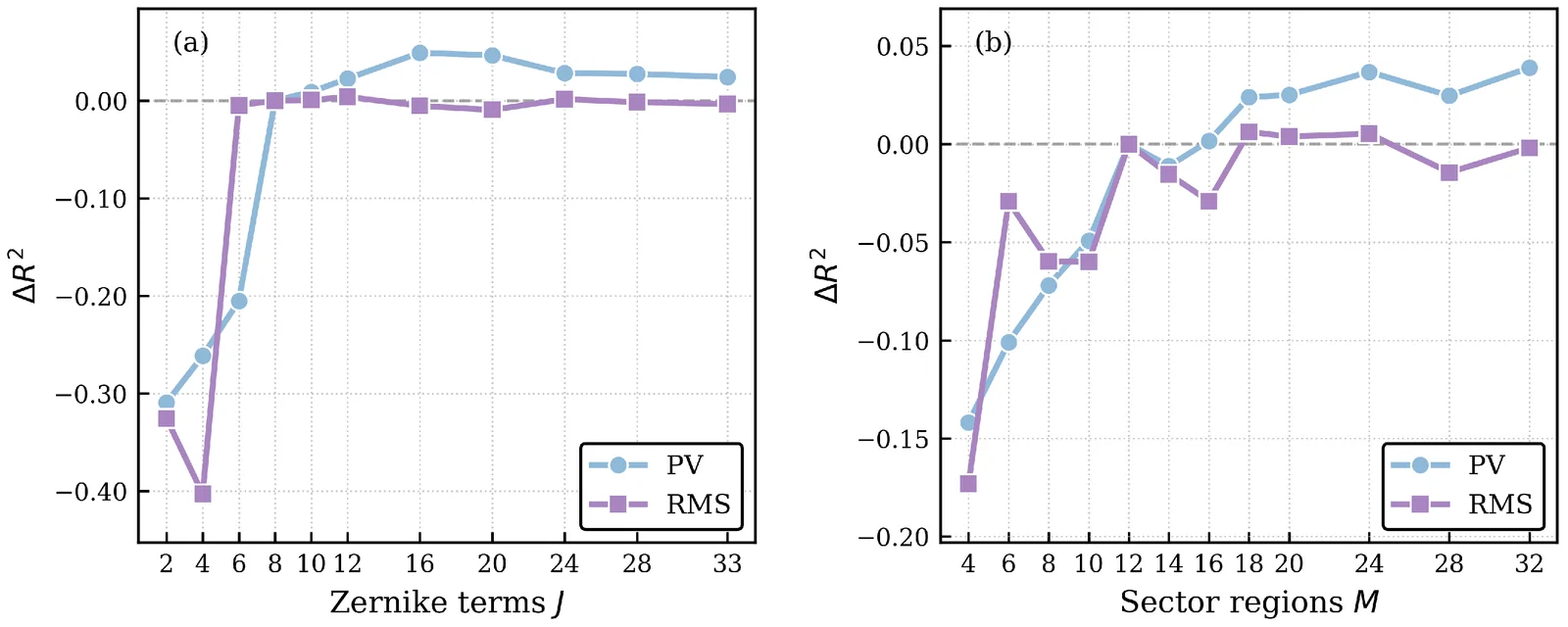
Sensitivity analysis of RPVF feature construction parameters. The vertical axis denotes the change in $R^{2}$ relative to the baseline setting, with the red zero line indicating no change from the baseline. (**a**) Results for different *J* values relative to J=8 at fixed M=12. (**b**) Results for different *M* values relative to M=12 at fixed J=8.

The main parameters in RPVF feature construction are the Zernike truncation term number *J* and the number of sector regions *M*. Specifically, *J* determines the number of surface figure modes retained in the low-order reconstructed wavefront, thereby affecting the low-order surface figure information contained in the regional fluctuation features. Meanwhile, *M* determines the angular resolution of the regional partition, thereby influencing the ability of the peak-to-valley fluctuation features to characterize local spatial variations. Since both parameters affect the information content and feature dimensionality of the augmented input, a parameter sensitivity analysis is further conducted in this study. Figure 4 shows the variations in $R^{2}$ under different parameter settings relative to the setting adopted in this study. Under this setting, the $R^{2}$ values for PV and RMS are 0.578 and 0.727, respectively.

As shown in Figure 4a, when J<8, the prediction performance for PV decreases significantly. As *J* continues to increase, the PV prediction performance improves only slightly, with a maximum increment of approximately 0.049, while the RMS prediction performance remains generally stable. This indicates that higher-order terms provide limited improvement in model performance. Therefore, J=8 is adopted as the Zernike truncation term number in the subsequent experiments, so as to balance the representation capability of low-order surface figure information and the complexity of auxiliary reconstruction.

As shown in Figure 4b, when M<12, the prediction performance for both PV and RMS generally decreases. As *M* further increases up to M=32, the maximum increment in PV is approximately 0.039, while RMS only exhibits slight fluctuations. Considering that a larger *M* continuously increases the dimensionality of the augmented input, M=12 is adopted as the number of sector regions in the subsequent experiments, so as to balance angular regional resolution, prediction performance, and model complexity.

Based on the above results, J=8 and M=12 are used as the RPVF feature construction parameters in the subsequent static ablation analysis, active-learning-based modeling, and tolerance-boundary inverse determination.

#### 3.2.2. Ablation Analysis of Feature Enhancement

**Figure 5:**
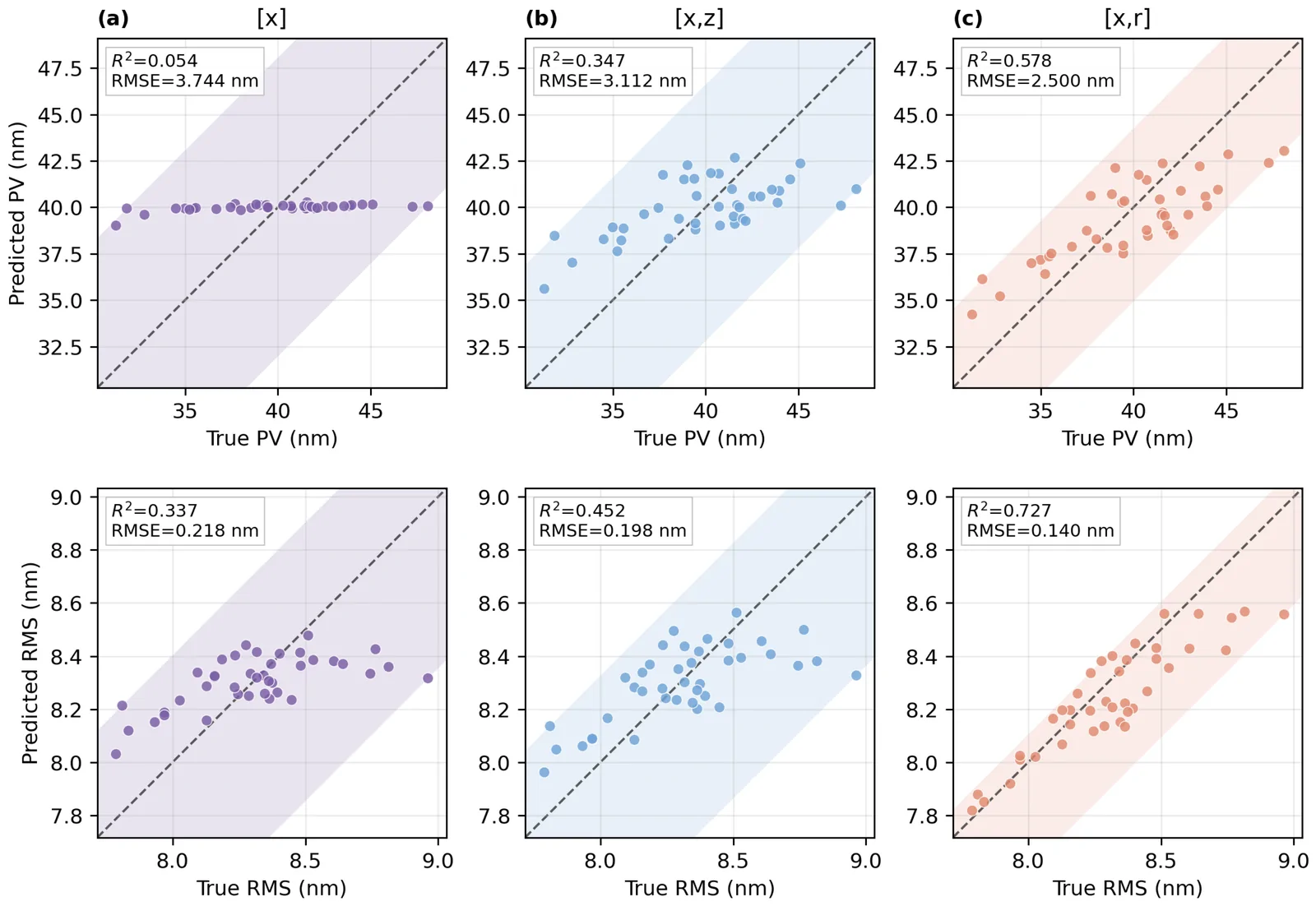
Comparison between true and predicted PV and RMS values under different static prediction schemes. The scatter points denote test samples, the dashed diagonal line represents the ideal prediction relationship, and the colored shaded regions enclose the scatter-point distribution, with a narrower region indicating smaller prediction errors: (**a**) x is the baseline scheme using only the support load inputs; (**b**) [x,z] is the joint-input scheme using support loads and low-order Zernike coefficients; (**c**) [x,r] is the joint-input scheme using support loads and RPVF features.

After the RPVF feature construction parameters are determined, static ablation experiments are conducted in this section to compare the relative effectiveness of different input representation schemes and to determine the input feature construction scheme for the subsequent active-learning-based modeling stage. Three types of input representation schemes are compared: the baseline scheme using only raw support loads x, the scheme of joint input of support loads and low-order Zernike coefficients [x,z], and the scheme of joint input of support loads and RPVF [x,r]. Among these, z represents the low-order Zernike coefficients predicted from support loads, and r represents the regional fluctuation features further extracted based on the low-order reconstructed wavefront. Static ablation results under different input representations are shown in Figure 5.

As shown in Figure 5, the diagonal line represents the ideal prediction relationship. Prediction accuracy is higher when the scatter points are closer to the diagonal and more tightly distributed. When only the support load vector x is used, the predicted PV and RMS values both deviate from the ideal diagonal. In particular, the predicted PV values are concentrated within a narrow range and do not vary consistently with the true PV values, resulting in $R^{2}$ values of 0.054 and 0.337 for PV and RMS, respectively. This indicates that under initial small sample conditions, the raw load vector has difficulty directly characterizing the complex surface shape response induced by load perturbations.

After adopting the joint input x,z, the prediction of both PV and RMS is improved, with the corresponding $R^{2}$ values increasing to 0.347 and 0.452, respectively. Compared with the baseline scheme, these represent increases of 0.293 and 0.115, indicating that the low-order Zernike coefficients provide additional global surface figure modal information to the load inputs. However, the scatter points obtained by directly concatenating z remain relatively dispersed. This suggests that a simple combination of low-order global modes and raw load variables is still insufficient to fully characterize regional peak-to-valley variations.

In contrast, when the joint input [x,r] is used, the scatter points for both PV and RMS move closer to the ideal diagonal. The $R^{2}$ values increase to 0.578 and 0.727, respectively, corresponding to improvements of 0.524 and 0.390 over the baseline scheme, while the RMSE values decrease to 2.500 nm and 0.140 nm, respectively. These results indicate that the regional peak-to-valley fluctuation features extracted from the low-order reconstructed wavefront can transform the global surface figure information contained in the Zernike modes into fluctuation descriptions at the spatial regional scale. In this way, they provide the model with local peak-to-valley response information that is difficult to express directly using the original load inputs. In particular, from the perspective of relative improvement, both PV and RMS are significantly improved compared with their respective baseline schemes, demonstrating that RPVF provides effective feature enhancement for both types of surface figure metrics.

The static ablation experiments in this section use the same initial training set, independent test set, and GPR settings, with a focus on investigating the influence of different input representations on the initial predictive capability of the model under limited-sample conditions. Under this setting, the [x,r] scheme achieves a higher $R^{2}$ and a lower RMSE, indicating that the RPVF features can provide a more effective initial model basis for subsequent active learning. Therefore, the joint input scheme [x,r] is adopted in the subsequent active-learning-based modeling and tolerance-boundary inverse determination. Meanwhile, certain prediction deviations still exist near some extreme-response samples in Figure 5, suggesting that the initial small-sample training set is still insufficient to fully cover the weak regions of the model. To further improve the prediction accuracy of the surrogate model within the target response range, an active learning strategy is subsequently introduced to supplement highly informative samples.

### 3.3. Active Learning Performance Analysis

**Figure 6:**
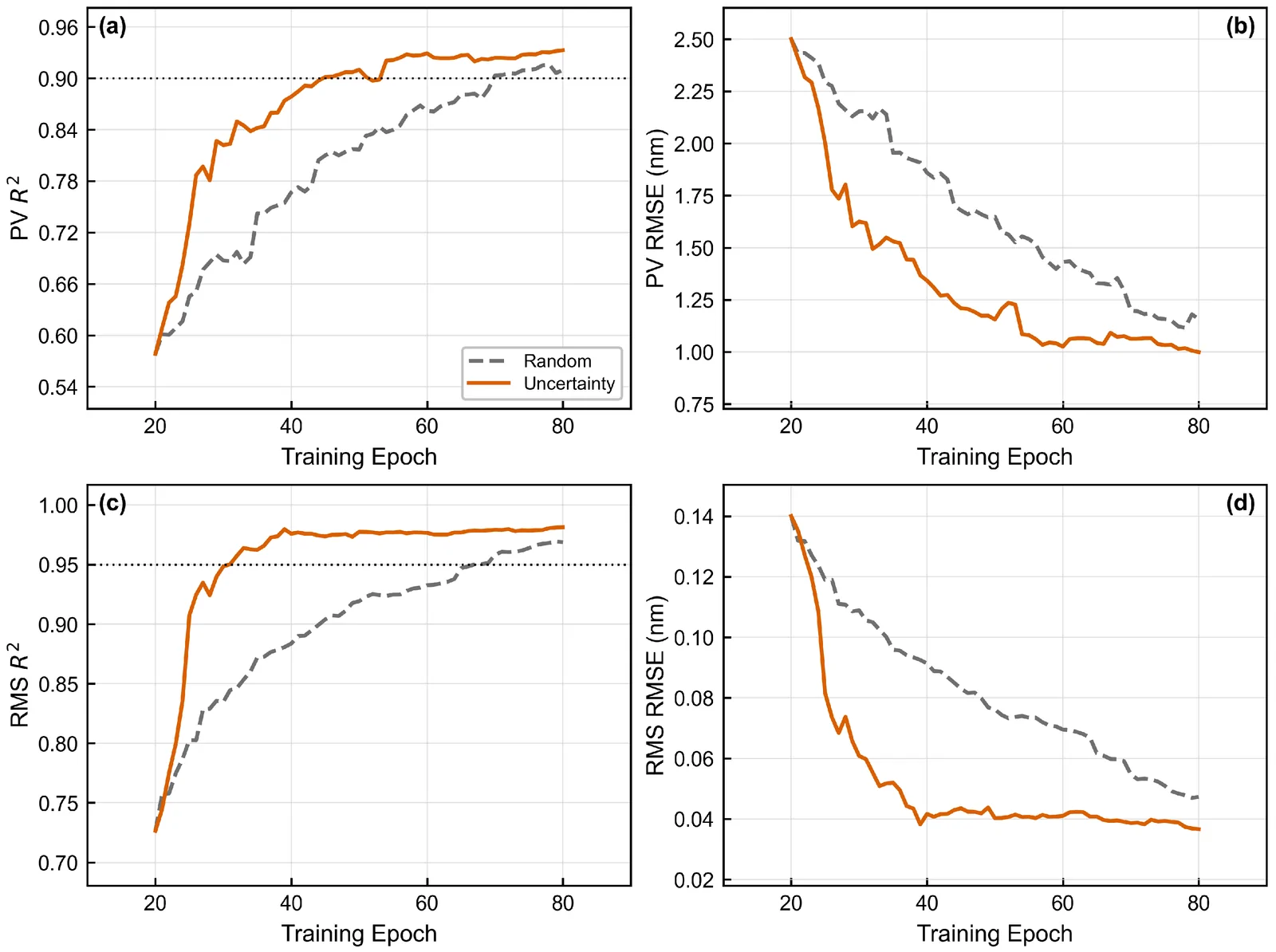
Comparison of training iteration processes between random sampling and uncertainty-driven sampling. (**a**,**b**) are the $R^{2}$ and RMSE changes in the PV indicator, respectively; (**c**,**d**) are the $R^{2}$ and RMSE changes in the RMS indicator, respectively.

To verify the effectiveness of the uncertainty-driven sampling strategy, it is compared with a random sampling baseline. In each iteration, random sampling selects a new sample randomly from the candidate pool, whereas uncertainty-driven sampling selects the sample to be added to the training set according to the combined posterior variance criterion defined in Equation (12), prioritizing the candidate with the highest predictive uncertainty under the current model. Both strategies use the same initial training set, candidate sample domain, and final sample budget. Figure 6 shows one representative training process obtained under this setting, which is used to compare the convergence trends and sample utilization efficiency of the two sampling strategies.

As shown in Figure 6, in this training run, uncertainty-driven sampling exhibits faster early-stage convergence for both the PV and RMS metrics. This strategy requires 45 and 31 samples to reach $\mathrm{PV}\;R^{2}\geq 0.90$ and $\mathrm{RMS}\;R^{2}\geq 0.95$, respectively, whereas random sampling requires 70 and 67 samples. This corresponds to reductions of 35.7% and 53.7%, indicating that the posterior variance criterion can preferentially add informative samples from regions where the current surrogate model is less well trained, thereby improving sample efficiency during active learning. In terms of final model accuracy, when the sample size reaches 80, the PV $R^{2}$ and RMS $R^{2}$ obtained by uncertainty-driven sampling are 0.933 and 0.981, respectively, compared with 0.910 and 0.969 obtained by random sampling. This corresponds to absolute improvements of 0.023 and 0.012 in $R^{2}$, respectively. Overall, this set of results indicates that, under the current experimental setting, uncertainty-driven sampling can accelerate early-stage convergence and achieve higher prediction accuracy under a fixed sample budget compared with random sampling.

In addition to prediction accuracy and sample efficiency, the stability of the surrogate model under input perturbations is also important for engineering applicability. To assess this stability, a model that has achieved high PV prediction accuracy during the active learning process is selected as the baseline, and random noise is added to the raw support load input of test samples during the inference phase. Noise forms include Gaussian noise and uniform noise, with noise levels set to 1%, 3%, 5%, and 10%. Each condition is evaluated 20 times repeatedly to simulate the impact of support load measurement errors, applied load deviations, or input parameter perturbations on model prediction results.

**Figure 7:**
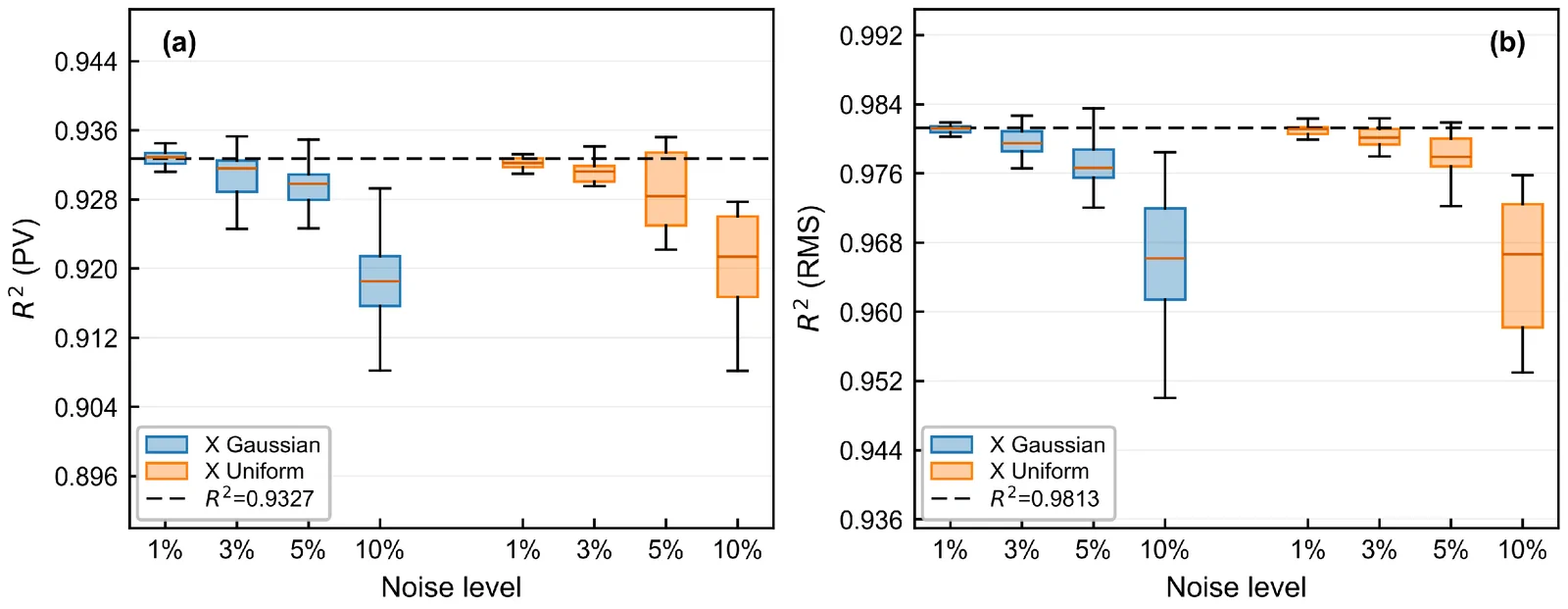
Box plots of prediction performance over repeated trials under input noise. The central line in each box denotes the median, and the black dashed line indicates the noise-free baseline value: (**a**) Distribution of $R^{2}$ for the PV metric at different noise levels; (**b**) Distribution of $R^{2}$ for the RMS metric at different noise levels.

**Figure 8:**
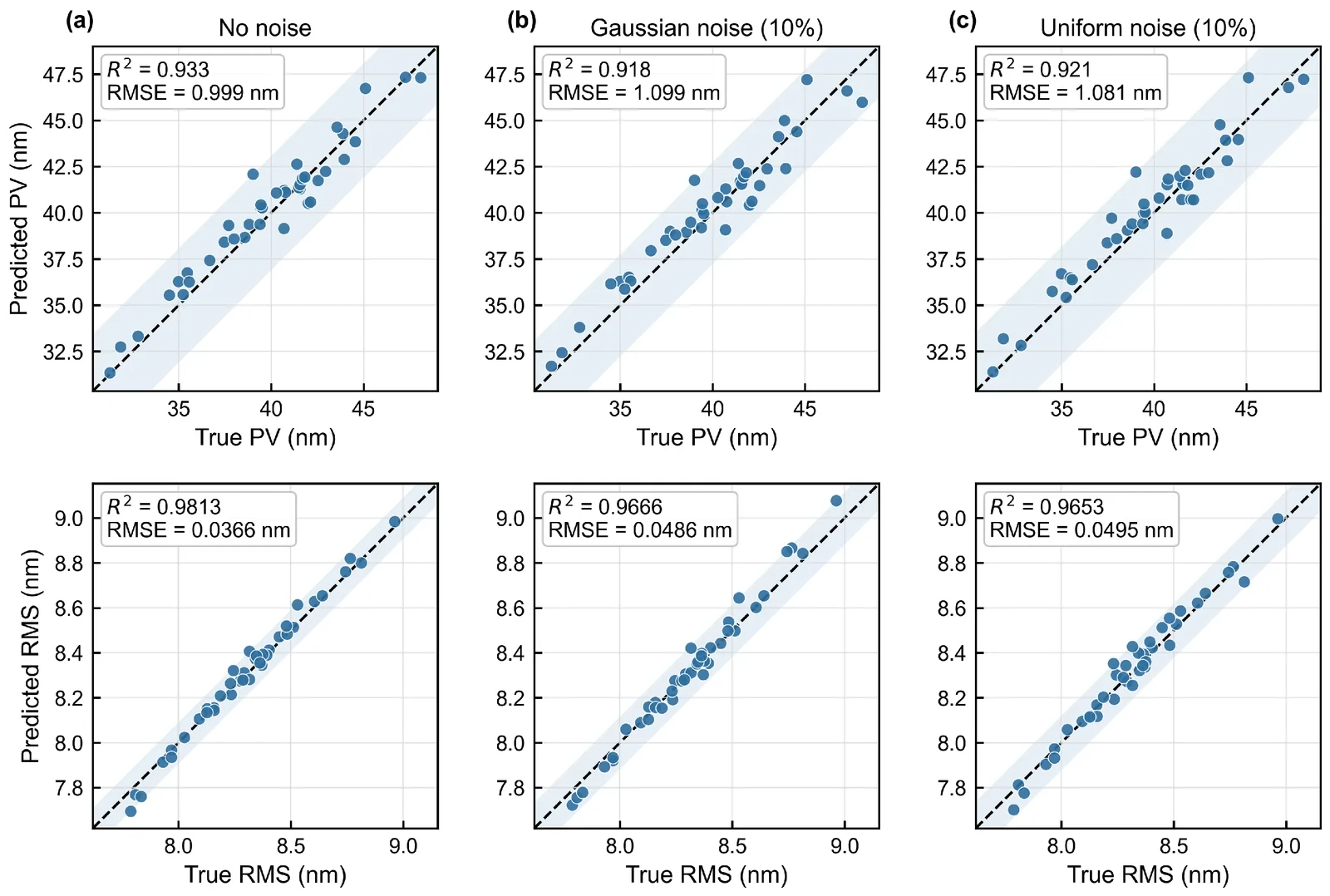
Comparison between predicted and true PV and RMS values under different input noise conditions. The scatter points denote test samples, the dashed diagonal line represents the ideal prediction relationship, and the shaded regions enclose the scatter-point distribution, with a narrower region indicating smaller prediction errors: (**a**) No noise; (**b**) 10% Gaussian noise; (**c**) 10% uniform noise.

Under the noise-free condition, the model achieves PV $R^{2}$ and RMS $R^{2}$ values of 0.9327 and 0.9813 on the test set, respectively. As shown in Figure 7, as the input noise level increases from 1% to 10%, the $R^{2}$ values of both metrics decrease gradually. At the highest noise level of 10%, the mean PV and RMS $R^{2}$ values under Gaussian noise are 0.9184 and 0.9666, corresponding to decreases of only 1.53% and 1.50%, respectively. Under uniform noise, the corresponding values are 0.9210 and 0.9653, with decreases of only 1.25% and 1.63%. Figure 8 further displays the prediction performance under 10% input noise. The scatter points remain mainly distributed near the ideal diagonal, indicating that the model maintains good prediction accuracy for both PV and RMS even under relatively large input perturbations. Overall, the input-enhanced GPR surrogate model after active learning limits the performance degradation to within 2% under 10% input perturbation, confirming its robustness in engineering applications and providing a stable forward prediction basis for subsequent support-load fluctuation tolerance inversion.

### 3.4. Tolerance Boundary Inversion and Verification

**Figure 9:**
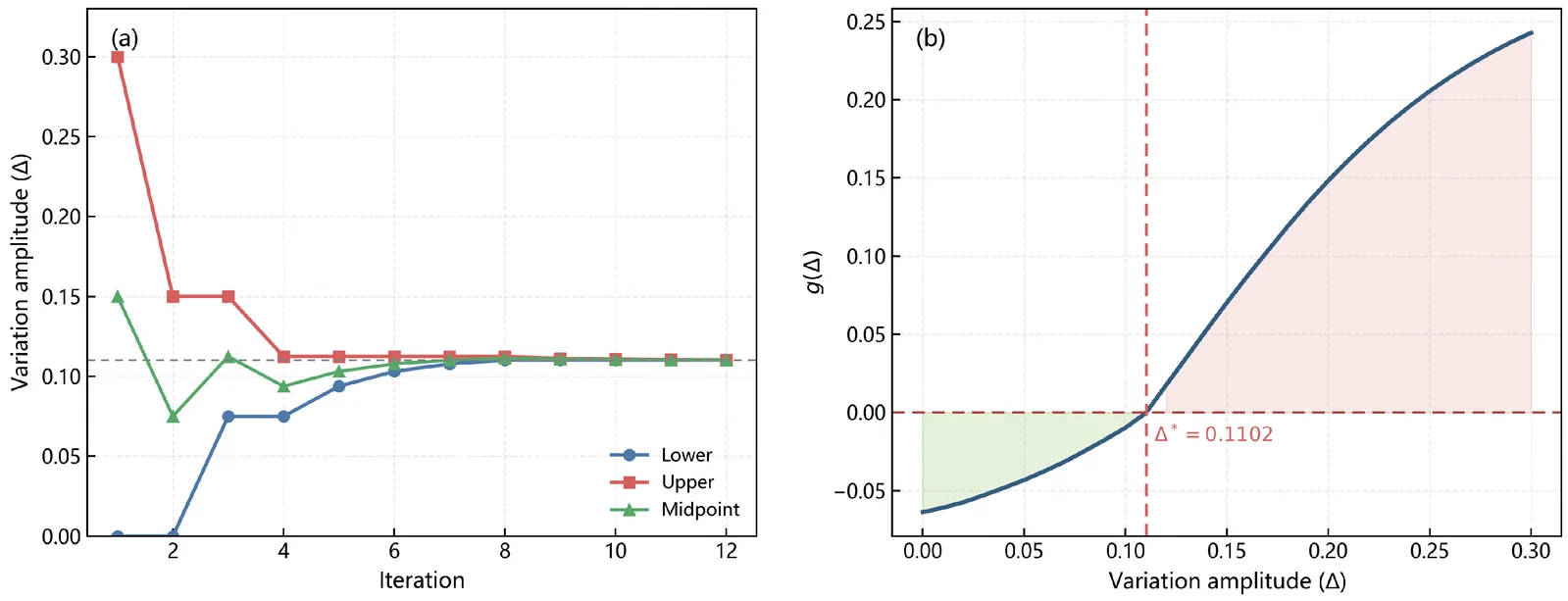
Search and localization process of the critical fluctuation boundary under dual-metric constraints. (**a**) Iterative evolution of the lower bound, upper bound, and midpoint of the fluctuation amplitude based on the bisection method. (**b**) Variation in the constraint function g(Δ) with the fluctuation amplitude Δ, where the green region denotes the feasible interval satisfying the constraints and the red region denotes the infeasible interval with predicted violation risk.

After completing active learning modeling, the final enhanced GPR model is used for support load fluctuation tolerance inversion. The dual-metric tolerance upper limits are set to ${\mathrm{PV}}_{\mathrm{tol}}=43.0\;\mathrm{nm}$ and ${\mathrm{RMS}}_{\mathrm{tol}}=8.6\;\mathrm{nm}$. The search and location process of the critical fluctuation boundary is shown in Figure 9. As the bisection search iteration advances, the search interval gradually contracts, and g(Δ) transitions from non-positive to positive values near the boundary. The final estimated critical fluctuation boundary is obtained as $\Delta^{*}=0.1102$, indicating that, under the current surrogate model and test sample setting, the estimated allowable fluctuation amplitude of the support loads relative to their theoretical values is approximately 11.02%. This result is obtained based on 3000 fixed test samples and should therefore be regarded as a boundary estimate under a finite-sample condition. Increasing the number of test samples can help improve the coverage of the load space and the stability of the boundary estimation, but it also increases the computational cost of prediction.

To verify the reliability of the estimated critical fluctuation boundary obtained from the inversion, three representative fluctuation levels near $\Delta^{*}$ are selected for finite element validation. If the inverted boundary is reasonable, fluctuation levels not exceeding $\Delta^{*}$ should satisfy the PV and RMS tolerance constraints, whereas levels beyond $\Delta^{*}$ should exhibit a risk of violation. For each fluctuation level, the constraint function r(x) is used to select a high-risk sample from the corresponding candidate load set as the validation case. The finite element validation results are listed in Table 2. When the fluctuation amplitude does not exceed $\Delta^{*}$, the constraint function remains negative, and both PV and RMS stay within their tolerance limits. When the fluctuation amplitude increases to $\Delta^{*}+0.02$, the constraint function becomes positive, and RMS exceeds its tolerance limit. These results indicate that the obtained $\Delta^{*}$ provides a reasonable estimate of the critical fluctuation boundary and can distinguish feasible cases from cases with violation risk under the prescribed PV and RMS constraints.

## 4. Conclusions

In summary, we constructed a Regional Peak-to-Valley Fluctuation Feature-enhanced Active-Learning Gaussian Process Regression framework for tolerance inversion. The framework first constructs surrogate mappings from the support loads to the low-order Zernike coefficients and extracts regional peak-to-valley fluctuation features from the low-order reconstructed wavefront, thereby transforming local surface figure fluctuation information into enhanced inputs for the PV and RMS surrogate models. The original loads and the extracted regional features are then jointly used as inputs to the Gaussian process regression models for PV and RMS. Posterior-uncertainty-driven active learning is adopted to guide sample selection and improve modeling efficiency under limited simulation samples. On this basis, a PV/RMS dual-metric constraint function is further constructed and combined with bisection search to estimate the critical support-load fluctuation boundary.

A biconvex lens support system was used as a case study. With the regional peak-to-valley fluctuation feature, the small-sample prediction $R^{2}$ values of PV and RMS increased by 0.524 and 0.390, respectively, compared with the baseline using only the original support loads. This confirms that the proposed feature can effectively supplement local peak-to-valley response information that is difficult to represent directly from raw load inputs. After active learning, the numbers of samples required to reach $\mathrm{PV}\;R^{2}\geq 0.90$ and $\mathrm{RMS}\;R^{2}\geq 0.95$ were reduced by 35.7% and 53.7%, respectively, compared with random sampling. With 80 samples, the final PV and RMS prediction $R^{2}$ values reached 0.933 and 0.981. Input perturbation tests showed that, under 10% Gaussian and uniform noise, the decreases in $R^{2}$ for both metrics remained within 2%, indicating good sample efficiency, prediction accuracy, and robustness. Furthermore, under the constraints ${\mathrm{PV}}_{\mathrm{tol}}=43.0\;\mathrm{nm}$ and ${\mathrm{RMS}}_{\mathrm{tol}}=8.6\;\mathrm{nm}$, the critical load fluctuation boundary based on a finite fixed test sample pool was estimated as $\Delta^{*}=0.1102$. Finite element validation showed that this boundary estimate can effectively distinguish feasible cases from cases with violation risk. These results provide a reference for support-load tolerance allocation, assembly control, and optomechanical stability evaluation in precision optical systems such as lithographic objectives.

## Figures and Tables

**Table 1:** Lens Material Parameters.

Parameter	Young’s Modulus, *E*	Poisson’s Ratio, ν	Density, ρ
Value	$9.03\times 10^{10}\;\mathrm{Pa}$	0.24	$2530\;\mathrm{kg}/m^{3}$

**Table 2:** Simulation verification results of the critical fluctuation amplitude.

Condition	Δ	PV (nm)	RMS (nm)	r(x)	Status
$\Delta^{*}-0.02$	0.0902	40.7686	8.3808	−0.0255	Feasible
$\Delta^{*}$	0.1102	41.7594	8.4893	−0.0129	Feasible
$\Delta^{*}+0.02$	0.1302	42.7503	8.6118	0.0014	Violation

## Data Availability

The original contributions presented in this study are included in the article. Further inquiries can be directed to the corresponding author.
